# Supplementary material for: Identification of Freezing-Responsive microRNAs and Their Targets in Chinese Jujube by Small RNA and Degradome Sequencing
Source: Int J Mol Sci. 2026 Sep 4;27(17):7912. doi: 10.3390/ijms27177912 (PMC13566890; doi:10.3390/ijms27177912)
Supplement: Supplementary file 1 [file ijms-27-07912-s001.zip › Supplementary Material S2/novel_miR_185.pdf]

Provisional ID	:	novel_miR_185
Score total	:	91550.5
Score for star read(s)	:	3.9
Score for read counts	:	91544.8
Score for mfe	:	0.1
Score for randfold	:	1.6
Score for cons. seed	:	
Total read count	:	179573
Mature read count	:	179050
Loop read count	:	3
Star read count	:	520

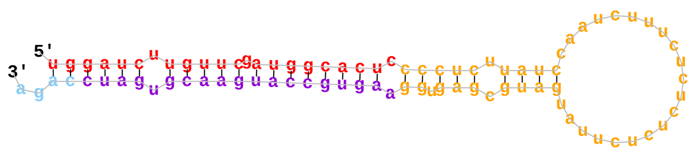

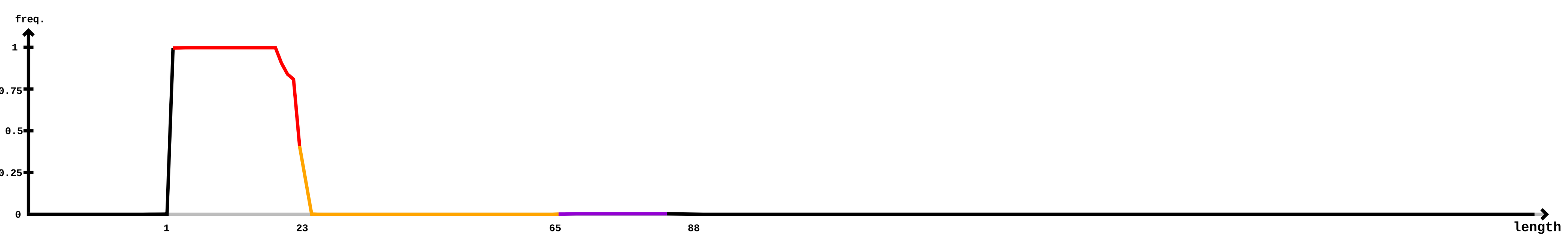

Mature Star

```
5'- uccaucuuguucguucacucucuggaucuuguucgauggcacuccccucuuauccaaucuuucucucucucuuaugaugcgaguggaagugccaugaacgugauccagaaugugcuuuccaacaaguuacaccuacuuaauaucuacuucuuuuuucuuuuuucuuuuuucuuccauuacuauuauuaaggaugguuucuuuuucugguucugauuuuuauuuacuuauuuauuuauuuguuuuuugugu -3' obs
    uccaucuuguucguucacucucuggaucuuguucgauggcacuccccucuuauccaaucuuucucucucucuuaugaugcgaguggaagugccaugaacgugauccagaaugugcuuuccaacaaguuacaccuacuuaauaucuacuucuuuuuucuuuuuucuuuuuucuuccauuacuauuauuaaggaugguuucuuuuucugguucugauuuuuauuuacuuauuuauuuauuuguuuuuugugu      exp
    .....((((((.(..(((..((((((((.(((((.((((((((.(((((.((((....................)))).))).)).))))))))))))).))))))))..)))....).))))))....(((..(((((((.....................................)))))))...)))...................................................... reads mm sample
    .................ucucuggaucuuguucgauggc.................................................................................................. 1 0 LT-3
    .................ucucuggaucuuguucgauggc.................................................................................................. 32 0 JCK-2
    .................ucucuggaucuuguucgauggc.................................................................................................. 22 0 JCK-3
    .................ucucuggaucuuguucgauggc.................................................................................................. 15 0 JCK-1
    .................ucucuggaucuuguucgauggcacu............................................................................................... 2 0 JCK-1
    .................ucucuggaucuuguucgauggcacu............................................................................................... 1 0 LT-3
    .................ucucuggaucuuguucgauNgcacu............................................................................................... 1 1 JCK-1
    ..................cucuggaucuuguuUgau...................................................................................................... 2 1 JCK-3
    ..................cucuggaucuuguucgauggc.................................................................................................. 3 0 JCK-2
    ..................cucuggaucuuguucgauggc.................................................................................................. 2 0 JCK-1
    ..................cucuggaucuuguucgauggc.................................................................................................. 1 0 JCK-3
    ..................cucuggaucuuguucgauggc.................................................................................................. 9 0 LT-3
    ..................cucuggaucuuguucgCuggc.................................................................................................. 2 1 LT-3
    ..................cucuggaucuuguucgauggc.................................................................................................. 1 0 LT-1
    ..................Aucuggaucuuguucgauggcacu............................................................................................... 1 1 JCK-1
    ...................ucuggaucuuguucgauggc.................................................................................................. 3 0 JCK-1
    ...................ucuggaucuuguucgauggc.................................................................................................. 1 0 JCK-2
    ...................ucuggaucuuguucgauggca................................................................................................. 1 0 JCK-3
    ...................ucuggaucuuguucgauggca................................................................................................. 1 0 JCK-1
    ...................ucuggaucuuguucgauggca................................................................................................. 4 0 LT-3
    ...................ucuggaucuuguucgauggca................................................................................................. 2 0 LT-2
    ...................ucuggaucuuguucgauAgca................................................................................................. 1 1 JCK-3
    ...................ucuggaucuuguucgauggcac................................................................................................ 1 0 LT-1
    ...................ucuggaucuuguucgauggcac................................................................................................ 1 0 JCK-2
    ...................ucuggaucuuguucgauggcac................................................................................................ 1 0 JCK-1
    ...................ucuggaucuuguucgauggcacuc.............................................................................................. 3 0 JCK-2
    ...................ucuggaucuuguucgauggcacuc.............................................................................................. 1 0 JCK-1
    ...................ucuggaucuuguucgauggcacuc.............................................................................................. 2 0 JCK-3
    ...................ucuggaucuuguucgauggcacuc.............................................................................................. 1 0 LT-3
    ....................cuggaucuuguucgauggc.................................................................................................. 1 0 JCK-1
    ....................cuggaucuuguucgauggc.................................................................................................. 3 0 LT-2
    ....................cuggaucuuguucgauggc.................................................................................................. 2 0 LT-1
    ....................cuggaucuuguucgauggca................................................................................................. 2 0 LT-1
```

```
                      Mature                                                          Star
uccaucuuguucguucacucucuggaucuuguucgauggcacuccccucuuauccaaucuuucucucucuuaugaugcgaguggaagugccaugaacgugauccagaaugugcuuuccaacaaguuacaccuacuuaauaucuacuucuuuuuucuuuuuucuuuuuucuuccauuacuauuauuaaggaugguuucuuuucugguucugauuuuuauuuacuuauuuauuuauuuguuuuuugugu
.....................cuggaucuuguucgauggcac.............................................................................................................................................................................................................   2      0   LCK-3
.....................cuggaucuuguucgauggcacu............................................................................................................................................................................................................   7      0   LT-3
.....................cuggaucuuguucgauggcacu............................................................................................................................................................................................................   8      0   JCK-3
.....................cuggaucuuguucgauggcacu............................................................................................................................................................................................................   6      0   JCK-1
.....................cuggaucuuguucgauggcacu............................................................................................................................................................................................................   16     0   JCK-2
.....................cuggaucuuguucgauggcacu............................................................................................................................................................................................................   5      0   LT-2
.....................cuggaucuuguucgauggcacu............................................................................................................................................................................................................   2      0   LT-1
.....................cuggaucuuguucgauggcacuU...........................................................................................................................................................................................................   1      1   LT-3
.....................cuggaucuuguucgauggcacuc...........................................................................................................................................................................................................   2      0   LT-1
.....................cuggaucuuguucgauggcacuc...........................................................................................................................................................................................................   3      0   LT-2
.....................cuggaucuuguucgauggcacuc...........................................................................................................................................................................................................   2      0   JCK-3
.....................cuggaucuuguucgauggcacuc...........................................................................................................................................................................................................   1      0   LT-3
.....................cuggaucuuguucgauggcacuc...........................................................................................................................................................................................................   3      0   JCK-1
.....................cuggaucuuguucgauggcacuc...........................................................................................................................................................................................................   4      0   JCK-2
......................uNgaucuuguucgauggc...............................................................................................................................................................................................................   1      1   JCK-2
......................uAgaucuuguucgauggc...............................................................................................................................................................................................................   1      1   LT-2
......................Cggaucuuguucgauggc...............................................................................................................................................................................................................   1      1   LCK-3
......................uggCucuuguucgauggc...............................................................................................................................................................................................................   2      1   LT-1
......................uggaucuuguucgauggc...............................................................................................................................................................................................................   1716   0   LT-3
......................uggaucuugCucgauggc...............................................................................................................................................................................................................   2      1   LT-2
......................uggaucuuguucgauggA...............................................................................................................................................................................................................   2      1   LT-2
......................uggaucuuguucgauggc...............................................................................................................................................................................................................   2116   0   LT-1
......................uggaucuugCucgauggc...............................................................................................................................................................................................................   1      1   JCK-2
......................uggaucuuguucgauggU...............................................................................................................................................................................................................   26     1   LCK-3
......................uggauAuuguucgauggc...............................................................................................................................................................................................................   1      1   LT-1
......................uggaucuuguucgGuggc...............................................................................................................................................................................................................   1      1   LT-1
......................uUgaucuuguucgauggc...............................................................................................................................................................................................................   2      1   LT-2
......................uggaucuuguucgauggc...............................................................................................................................................................................................................   186    0   JT-1
......................uggaucuuguucgauggc...............................................................................................................................................................................................................   5148   0   LCK-3
......................uggCucuuguucgauggc...............................................................................................................................................................................................................   1      1   JCK-2
......................uggaucuuguucgauAgc...............................................................................................................................................................................................................   1      1   LCK-3
......................uggaucuuguucgauggc...............................................................................................................................................................................................................   182    0   JT-3
......................uggGucuuguucgauggc...............................................................................................................................................................................................................   1      1   LCK-3
......................uggaucuugCucgauggc...............................................................................................................................................................................................................   1      1   LT-1
......................uggaucuuguucgauggc...............................................................................................................................................................................................................   261    0   JCK-3
......................uggaucuuguucgauggc...............................................................................................................................................................................................................   253    0   JCK-1
......................uggaucuuguucgCuggc...............................................................................................................................................................................................................   2      1   LCK-1
......................uggaucuuguucUauggc...............................................................................................................................................................................................................   1      1   LT-1
......................uggaucuuguucUauggc...............................................................................................................................................................................................................   3      1   LT-2
......................uggaucuuguucgauCgc...............................................................................................................................................................................................................   1      1   LCK-3
......................uggaucuugCucgauggc...............................................................................................................................................................................................................   1      1   LT-3
......................uggaucuuguucgauggU...............................................................................................................................................................................................................   1      1   JCK-3
......................uggaucuuguucgCuggc...............................................................................................................................................................................................................   1      1   JT-2
......................uggaucuuguucgauggA...............................................................................................................................................................................................................   6      1   LT-3
......................uggaucuuguucgaugAc...............................................................................................................................................................................................................   4      1   LCK-3
......................uggaucuuAuucgauggc...............................................................................................................................................................................................................   1      1   LCK-3
......................uggCucuuguucgauggc...............................................................................................................................................................................................................   2      1   LT-3
......................uggaucuuguucgCuggc...............................................................................................................................................................................................................   5      1   LT-3
......................uggaucuuguucgCuggc...............................................................................................................................................................................................................   19     1   LT-2
......................uAgaucuuguucgauggc...............................................................................................................................................................................................................   1      1   LT-1
......................uggaucuuguucgNuggc...............................................................................................................................................................................................................   2      1   LCK-1
......................uggaucuuguucgCuggc...............................................................................................................................................................................................................   1      1   LCK-2
......................uggaucuuguucgauggc...............................................................................................................................................................................................................   377    0   JCK-2
......................uggaucuuguucgauggc...............................................................................................................................................................................................................   134    0   JT-2
......................uggaucuuguucgauggA...............................................................................................................................................................................................................   2      1   JCK-3
......................uggaucuuguucgaugCc...............................................................................................................................................................................................................   1      1   LCK-3
......................ugAaucuuguucgauggc...............................................................................................................................................................................................................   1      1   LCK-3
......................uggaucuuguucgCuggc...............................................................................................................................................................................................................   1      1   JT-3
......................uggaucuuguucgGuggc...............................................................................................................................................................................................................   1      1   LCK-1
......................uggaCcuuguucgauggc...............................................................................................................................................................................................................   1      1   LT-2
......................uggaucCuguucgauggc...............................................................................................................................................................................................................   1      1   LT-2
......................uggaucuuguucgCuggc...............................................................................................................................................................................................................   8      1   LCK-3
......................uggCucuuguucgauggc...............................................................................................................................................................................................................   37     1   LT-2
......................uggaucuuguucgaugUc...............................................................................................................................................................................................................   1      1   LT-2
......................uggaucCuguucgauggc...............................................................................................................................................................................................................   1      1   LCK-3
......................uggaucuuguucgauggN...............................................................................................................................................................................................................   1      1   JCK-3
......................uAgaucuuguucgauggc...............................................................................................................................................................................................................   2      1   LT-3
......................ugAaucuuguucgauggc...............................................................................................................................................................................................................   1      1   LT-1
......................uggaucuuguucgauggc...............................................................................................................................................................................................................   4850   0   LT-2
......................uggaucuuguucgauggc...............................................................................................................................................................................................................   489    0   LCK-2
```

```
                      Mature                                                                          Star
uccaucuuguucguucacucucuggaucuuguucgauggcacuccccucuuauccaaucuuucucucucucuuaugaugcgaguggaagugccaugaacgugauccagaaugugcuuuccaacaaguuacaccuacuuaauaucuacuucuuuuuucuuuuuucuuuuuucuuccauuacuauuauuaaggaugguuucuuuuucugguucugauuuuuauuuacuuauuuauuuauuuguuuuuugugu
......................ugAaucuuguucgauggc........................................................................................................................................................................................................   1     1   LCK-2
......................uggaucuuguucgauAgc........................................................................................................................................................................................................   1     1   LT-2
......................uggaucuuguucgauggc........................................................................................................................................................................................................   522   0   LCK-1
......................uggCucuuguucgauggc........................................................................................................................................................................................................   9     1   LCK-3
......................uggaucuuguCcgauggc........................................................................................................................................................................................................   3     1   JCK-2
......................uggaucuuguucgauAgc........................................................................................................................................................................................................   1     1   JCK-1
......................uggaucuuguucgCuggc........................................................................................................................................................................................................   7     1   LT-1
......................uAgaucuuguucgauggc........................................................................................................................................................................................................   5     1   LCK-3
......................ugNaucuuguucgauggc........................................................................................................................................................................................................   13    1   LT-2
......................uggaucuuguucgaugAc........................................................................................................................................................................................................   3     1   LT-2
......................uggauUuuguucgauggc........................................................................................................................................................................................................   1     1   LT-2
......................uNgaucuuguucgauggc........................................................................................................................................................................................................   1     1   JCK-3
......................uggaucuuguucgauggG........................................................................................................................................................................................................   2     1   LT-3
......................uggaucuuguucgaCggc........................................................................................................................................................................................................   1     1   JCK-2
......................uggGucuuguucgauggc........................................................................................................................................................................................................   1     1   LT-1
......................ugAaucuuguucgauggc........................................................................................................................................................................................................   1     1   LT-2
......................uggaucuuguucAauggc........................................................................................................................................................................................................   1     1   LT-2
......................uggaucuuguucAauggc........................................................................................................................................................................................................   1     1   LCK-3
......................uggaucuuguuNgauggc........................................................................................................................................................................................................   2     1   JCK-1
......................uggaucuuguucgauggU........................................................................................................................................................................................................   3     1   LT-2
......................uAgaucuuguucgauggc........................................................................................................................................................................................................   1     1   LCK-2
......................uggaucuuguuUgauggc........................................................................................................................................................................................................   1     1   LT-1
......................uggaucuuguucgauggU........................................................................................................................................................................................................   22    1   LCK-2
......................uggaucuuguucgauggU........................................................................................................................................................................................................   23    1   LCK-1
......................ugAaucuuguucgauggc........................................................................................................................................................................................................   1     1   LT-3
......................uggaucuuguucgauggU........................................................................................................................................................................................................   5     1   LT-1
......................uggaucuuguucgauggA........................................................................................................................................................................................................   2     1   LCK-3
......................ugCaucuuguucgauggc........................................................................................................................................................................................................   2     1   LT-2
......................uggaucuuguucgauggU........................................................................................................................................................................................................   7     1   LT-3
......................uggaucuuguucgaNggc........................................................................................................................................................................................................   6     1   LCK-1
......................uggaucuuguucgCuggca.......................................................................................................................................................................................................   1     1   LT-3
......................uggNucuuguucgauggca.......................................................................................................................................................................................................   1     1   JCK-2
......................uAgaucuuguucgauggca.......................................................................................................................................................................................................   1     1   LCK-3
......................uggaucuuguuUgauggca.......................................................................................................................................................................................................   1     1   JT-1
......................Cggaucuuguucgauggca.......................................................................................................................................................................................................   1     1   JCK-2
......................uggaucuuguucgaugAca.......................................................................................................................................................................................................   2     1   LT-1
......................uggaucuuguGcgauggca.......................................................................................................................................................................................................   1     1   JCK-1
......................uggaucuuguucgauggcU.......................................................................................................................................................................................................   1     1   JCK-3
......................ugAaucuuguucgauggca.......................................................................................................................................................................................................   1     1   LCK-2
......................uggaucuuguucgaGggca.......................................................................................................................................................................................................   1     1   LCK-1
......................uggGucuuguucgauggca.......................................................................................................................................................................................................   1     1   LCK-1
......................uggaucuuAuucgauggca.......................................................................................................................................................................................................   1     1   LT-1
......................uggCucuuguucgauggca.......................................................................................................................................................................................................   7     1   LCK-3
......................uggaucuuguucAauggca.......................................................................................................................................................................................................   1     1   LCK-1
......................ugAaucuuguucgauggca.......................................................................................................................................................................................................   1     1   LT-2
......................uggaucuuguucgauggcC.......................................................................................................................................................................................................   1     1   LT-1
......................uggaucuuguucgauggcU.......................................................................................................................................................................................................   13    1   LCK-3
......................uggaucuuguucgauggcC.......................................................................................................................................................................................................   1     1   LCK-1
......................uggaucuuguucgauggca.......................................................................................................................................................................................................   1906  0   LT-2
......................uggaucuuguucgauggcU.......................................................................................................................................................................................................   8     1   JCK-2
......................uggaucuuguucgauggca.......................................................................................................................................................................................................   411   0   JCK-2
......................uAgaucuuguucgauggca.......................................................................................................................................................................................................   1     1   LCK-2
......................uggaucuuguucgauggcU.......................................................................................................................................................................................................   20    1   LCK-2
......................uggaucuuguucgCuggca.......................................................................................................................................................................................................   1     1   JCK-1
......................uggaucuuguucgauggca.......................................................................................................................................................................................................   259   0   JCK-1
......................uggaucuuguuAgauggca.......................................................................................................................................................................................................   1     1   LCK-2
......................uggaucuuguucgGuggca.......................................................................................................................................................................................................   1     1   LCK-1
......................uggaucuuguucgauggcG.......................................................................................................................................................................................................   1     1   LT-1
......................uggauNuuguucgauggca.......................................................................................................................................................................................................   2     1   LCK-1
......................uggaucuuguucgauggcG.......................................................................................................................................................................................................   8     1   LCK-3
......................uggaucuuUuucgauggca.......................................................................................................................................................................................................   1     1   LT-2
......................uggaucuuAuucgauggca.......................................................................................................................................................................................................   1     1   LT-2
......................ugAaucuuguucgauggca.......................................................................................................................................................................................................   1     1   LCK-3
......................uggaucuuguucgauggca.......................................................................................................................................................................................................   2503  0   LCK-3
......................uggCucuuguucgauggca.......................................................................................................................................................................................................   10    1   LT-2
......................uggaucuuguucgauggcG.......................................................................................................................................................................................................   2     1   JT-3
......................uggaucuuguucgauAgca.......................................................................................................................................................................................................   4     1   JCK-2
......................uggaucuuguucgCuggca.......................................................................................................................................................................................................   2     1   LCK-1
......................uggaucuuguucgauggca.......................................................................................................................................................................................................   182   0   JT-2
......................uggCucuuguucgauggca.......................................................................................................................................................................................................   2     1   LT-3
```

```
                      Mature                                                          Star
uccaucuuguucguucacucucuggaucuuguucgauggcacuccccucuuauccaaucuuucucucucucuuaugaugcgaguggaagugccaugaacgugauccagaaugugcuuuccaacaaguuacaccuacuuaauaucuacuucuuuuuucuuuuuucuuuuuucuuccauuacuauuauuaaggaugguuucuuuuucugguucugauuuuuauuuacuuauuuauuuauuuguuuuuugugu
......................uggaucuuguucgauAgca.................................................................................................................................................................................................................    4     1   JCK-1
......................uggaucuuguucgauggcU.................................................................................................................................................................................................................    7     1   JT-3
......................uggaucuuguucgauggca.................................................................................................................................................................................................................    1101  0   LT-1
......................uggaucuuguucgCuggca.................................................................................................................................................................................................................    1     1   JCK-2
......................uggCucuuguucgauggca.................................................................................................................................................................................................................    2     1   JCK-1
......................uggaucuuguucgauggcC.................................................................................................................................................................................................................    2     1   LCK-3
......................uggCucuuguucgauggca.................................................................................................................................................................................................................    7     1   LCK-2
......................uggaucuuguucgauggUa.................................................................................................................................................................................................................    1     1   LCK-3
......................uggaucuuguucgauggcC.................................................................................................................................................................................................................    5     1   LT-2
......................uggaucuuAuucgauggca.................................................................................................................................................................................................................    1     1   LT-3
......................uggaucuuguucgauggca.................................................................................................................................................................................................................    281   0   JT-3
......................uggaucuuguucgauggca.................................................................................................................................................................................................................    1208  0   LT-3
......................uggCucuuguucgauggca.................................................................................................................................................................................................................    1     1   LT-1
......................uggaucuuguucgauAgca.................................................................................................................................................................................................................    1     1   LT-2
......................uggaucuuguucgauggca.................................................................................................................................................................................................................    1539  0   LCK-1
......................uggaucuuguucgauggcU.................................................................................................................................................................................................................    24    1   LT-3
......................uAgaucuuguucgauggca.................................................................................................................................................................................................................    2     1   LT-2
......................uAgaucuuguucgauggca.................................................................................................................................................................................................................    2     1   LT-3
......................uggaucuuguucNauggca.................................................................................................................................................................................................................    1     1   LCK-1
......................uggaucuuguucgCuggca.................................................................................................................................................................................................................    2     1   LCK-3
......................uggaucuCguucgauggca.................................................................................................................................................................................................................    1     1   LCK-3
......................uggaucuuguucgauAgca.................................................................................................................................................................................................................    1     1   JCK-3
......................uggaucuuguucgauggcG.................................................................................................................................................................................................................    1     1   JCK-1
......................uggaucuuguucgNuggca.................................................................................................................................................................................................................    7     1   LCK-1
......................uggaucuuguucgauggcU.................................................................................................................................................................................................................    2     1   JT-2
......................uggaucuuguucgauggcC.................................................................................................................................................................................................................    1     1   JT-1
......................ugAaucuuguucgauggca.................................................................................................................................................................................................................    2     1   LCK-1
......................uggaucuuguucgauAgca.................................................................................................................................................................................................................    2     1   LCK-2
......................uggaucuuguucgauggAa.................................................................................................................................................................................................................    2     1   LT-1
......................uggaucuuguucgauggca.................................................................................................................................................................................................................    377   0   JT-1
......................uggaucuuguucgCuggca.................................................................................................................................................................................................................    3     1   LCK-2
......................uggaucuuguucUauggca.................................................................................................................................................................................................................    1     1   LT-3
......................uggaucuuguCcgauggca.................................................................................................................................................................................................................    1     1   LCK-3
......................uggaucuuguucgauggcC.................................................................................................................................................................................................................    1     1   LT-3
......................uggaucuuguucUauggca.................................................................................................................................................................................................................    6     1   LCK-3
......................uggaucuuguucgGuggca.................................................................................................................................................................................................................    1     1   LT-3
......................uggaucuuguucgauggcU.................................................................................................................................................................................................................    11    1   LT-1
......................uggaucuuguucUauggca.................................................................................................................................................................................................................    3     1   LT-2
......................uggCucuuguucgauggca.................................................................................................................................................................................................................    3     1   LCK-1
......................uggaucuuguucgauggcU.................................................................................................................................................................................................................    24    1   LCK-1
......................uggaucuuguucgauggca.................................................................................................................................................................................................................    306   0   JCK-3
......................uggGucuuguucgauggca.................................................................................................................................................................................................................    1     1   LCK-3
......................uggaucuuAuucgauggca.................................................................................................................................................................................................................    1     1   LCK-2
......................uggaucuuguucgauggca.................................................................................................................................................................................................................    1462  0   LCK-2
......................uggaucuuguucgCuggca.................................................................................................................................................................................................................    10    1   LT-2
......................ugNaucuuguucgauggca.................................................................................................................................................................................................................    1     1   LT-2
......................uggaucuuguucgauggcG.................................................................................................................................................................................................................    1     1   LT-3
......................uggaucuuguucgaNggca.................................................................................................................................................................................................................    2     1   LCK-1
......................uggaucuuguucgaCggca.................................................................................................................................................................................................................    1     1   JT-1
......................uggaucuuguucgauggcU.................................................................................................................................................................................................................    21    1   LT-2
......................uggaucuCguucgauggca.................................................................................................................................................................................................................    1     1   LT-2
......................uggaucuuguucgauggcCc................................................................................................................................................................................................................    1     1   LT-1
......................uggCucuuguucgauggcac................................................................................................................................................................................................................    1     1   JCK-1
......................uggaucuuguucgauggcUc................................................................................................................................................................................................................    6     1   LCK-3
......................uggaucuuguucgaCggcac................................................................................................................................................................................................................    1     1   LT-2
......................uggaucuuguucgauggcaA................................................................................................................................................................................................................    2     1   LT-1
......................uggaucuuguucgaCggcac................................................................................................................................................................................................................    1     1   LT-1
......................uggaucuuguucgauAgcac................................................................................................................................................................................................................    3     1   JCK-2
......................uggaucuuguucgauggcac................................................................................................................................................................................................................    108   0   JT-1
......................uggCucuuguucgauggcac................................................................................................................................................................................................................    1     1   LT-1
......................uggNucuuguucgauggcac................................................................................................................................................................................................................    1     1   LCK-1
......................uggaucuuguucgauggcaU................................................................................................................................................................................................................    26    1   LT-3
......................uggaucuuguucgauggcac................................................................................................................................................................................................................    40    0   JT-3
......................uggaucuuguucgauAgcac................................................................................................................................................................................................................    1     1   LT-2
......................uggaucuuguucUauggcac................................................................................................................................................................................................................    1     1   JT-1
......................uggaucuuguucgauggcac................................................................................................................................................................................................................    427   0   LCK-1
......................uggaCcuuguucgauggcac................................................................................................................................................................................................................    1     1   LT-2
......................uggaucuuguucgauggcac................................................................................................................................................................................................................    571   0   LT-1
......................uAgaucuuguucgauggcac................................................................................................................................................................................................................    1     1   LCK-3
......................Cggaucuuguucgauggcac................................................................................................................................................................................................................    1     1   LT-3
```

Mature **Star**

uccaucuuguucguucacucucuggaucuuguucgauggcacucccucuuauccaaucuuucucucucucuuaugaugcgaguggaagugccaugaacgugauccagaaugugcuuuccaacaaguuacaccuacuuaauaucuacuucuuuuuucuuuuuucuuuuuucuuccauuacuauuauuaaggaugguuucuuuuucugguucugauuuuuauuuacuuauuuauuuauuuguuuuuugugu

......................uggaucuuguucgauggcac......	122	0	JCK-1
......................uggaucuuguucgauggcac......	19	0	JT-2
......................uggaucuuguucgCuggcac......	1	1	LCK-3
......................uggaucuuguucgCuggcac......	1	1	LCK-1
......................uggaucuuguucgauggcac......	427	0	LCK-2
......................uggaucuCguucgauggcac......	1	1	LT-2
......................uggaucuuguuUgauggcac......	1	1	LT-1
......................uggaucuuguucgaugAcac......	1	1	LT-2
......................uggCucuuguucgauggcac......	1	1	LT-3
......................uggaucuuguucgaCggcac......	1	1	JCK-2
......................uggaucuuguucgauggcaU......	13	1	LT-2
......................uggaucuuguucgauggcCc......	1	1	LT-3
......................uggaucuuguucgauggcaU......	1	1	JT-2
......................uggaucuuguucgauAgcac......	1	1	JCK-1
......................uggaucuuguucgauggcaU......	4	1	JCK-3
......................uggaucuuguucgNuggcac......	2	1	LCK-1
......................uAgaucuuguucgauggcac......	1	1	LT-2
......................uggaucuuguucgauggcac......	118	0	JCK-3
......................uggaucuuguucgauAgcac......	3	1	JCK-3
......................uggaucuuguucgaGggcac......	1	1	LCK-1
......................uggaucuuguucgCuggcac......	1	1	LT-1
......................uggaucuuguucgauggcGc......	1	1	LT-2
......................uggaucuuguucgauggcac......	916	0	LT-2
......................uggaucuuguucgauggcaU......	13	1	LT-1
......................uggaucuuguucgauggcCc......	1	1	LCK-3
......................uggaucuuguucgauggcCc......	1	1	LT-2
......................uggaucuuguucgCuggcac......	3	1	LT-2
......................uggaucuuguucgauggcaA......	7	1	LT-2
......................uggaucuuguucgaNggcac......	1	1	LCK-1
......................uggCucuuguucgauggcac......	3	1	LCK-3
......................uggaucuuguucgCuggcac......	2	1	LT-3
......................uggaucCuguucgauggcac......	1	1	LT-3
......................ugNaucuuguucgauggcac......	4	1	LT-2
......................uggaucuuguucgauggcaG......	1	1	JCK-1
......................uggaucuuguucgauggcaU......	3	1	JT-3
......................uggaucuuguucgauggcac......	183	0	JCK-2
......................uggaucuuguucgCuggcac......	1	1	JCK-1
......................uggaucuuguucgauggcaU......	12	1	LCK-3
......................uggaucuuCuucgauggcac......	1	1	LT-2
......................uggaucuuguucgauggcUc......	1	1	LT-2
......................uggaucuuguucgauggcac......	1673	0	LCK-3
......................uggCucuuguucgauggcac......	1	1	JCK-3
......................uggCucuuguucgauggcac......	3	1	LT-2
......................ugAaucuuguucgauggcac......	1	1	JCK-3
......................uggaucuuguucgauggcaU......	3	1	JCK-1
......................uggaucuuguucgauggcac......	704	0	LT-3
......................uggaucuuguuUgauggcac......	1	1	LCK-3
......................uggaucuuguucAauggcacu......	3	1	LT-2
......................uggaucuuguucgauggcacA......	34	1	LCK-1
......................uggaucuugCucgauggcacu......	1	1	JCK-1
......................uggaucuuguucgaNggcacu......	5	1	LCK-1
......................uAgaucuuguucgauggcacu......	3	1	LCK-3
......................uggaucuuguuUgauggcacu......	4	1	LT-3
......................uggauUuuguucgauggcacu......	3	1	JCK-2
......................uggaucuuguucgauggcacA......	24	1	LCK-2
......................uUgaucuuguucgauggcacu......	2	1	JCK-3
......................uggaucuuguucgauggcaAu......	8	1	JCK-2
......................uggaucuuguGcgauggcacu......	2	1	JCK-1
......................uggaucuuguucgauggcacG......	5	1	LT-1
......................uggaCcuuguucgauggcacu......	2	1	LT-1
......................uggaucuuguucgauAgcacu......	12	1	JCK-1
......................uggCucuuguucgauggcacu......	5	1	LCK-2
......................uggaucuuguucgauggUacu......	6	1	JCK-2
......................uggaucuuguucgauggcacA......	12	1	LT-1
......................uggaucuuguucgauggcaUu......	10	1	LCK-3
......................uggaCcuuguucgauggcacu......	2	1	JCK-3
......................uggGucuuguucgauggcacu......	2	1	LT-3
......................uggaucuuguucgauggcUcu......	1	1	JT-2
......................uggGucuuguucgauggcacu......	1	1	JCK-2
......................uggaucuuguucgauggcacN......	1	1	JCK-1

```
                      Mature                                                          Star
uccaucuuguucguucacucucuggaucuuguucgauggcacuccccucuuauccaaucuuucucucucucuuaugaugcgaguggaagugccaugaacgugauccagaaugugcuuuccaacaaguuacaccuacuuaauaucuacuucuuuuuucuuuuuucuuuuuucuuccauuacuauuauuaaggaugguuucuuuuucugguucugauuuuuauuuacuuauuuauuuauuuguuuuuugugu
......................uAgaucuuguucgauggcacu...............................................................................................................................................................................   1      1     LCK-2
......................uggaucuuguucNauggcacu...............................................................................................................................................................................   1      1     LCK-1
......................uggaucuuguCcgauggcacu...............................................................................................................................................................................   1      1     LT-3
......................uggaucuuguucAauggcacu...............................................................................................................................................................................   12     1     JCK-2
......................uAgaucuuguucgauggcacu...............................................................................................................................................................................   2      1     JCK-2
......................uggaucuuguucgCuggcacu...............................................................................................................................................................................   3      1     LCK-2
......................uggaCcuuguucgauggcacu...............................................................................................................................................................................   1      1     LT-2
......................uggaucuuguucgaugAcacu...............................................................................................................................................................................   8      1     LT-1
......................uggCucuuguucgauggcacu...............................................................................................................................................................................   1      1     JT-1
......................uNgaucuuguucgauggcacu...............................................................................................................................................................................   6      1     JCK-3
......................uggaucuuguuUgauggcacu...............................................................................................................................................................................   1      1     JCK-1
......................uggNucuuguucgauggcacu...............................................................................................................................................................................   10     1     JCK-1
......................uggaucuuUuucgauggcacu...............................................................................................................................................................................   2      1     LT-3
......................uggaucuuguucgauggcacA...............................................................................................................................................................................   31     1     JCK-2
......................uggaucuuguucgaugAcacu...............................................................................................................................................................................   5      1     LT-3
......................uCgaucuuguucgauggcacu...............................................................................................................................................................................   1      1     JCK-2
......................uggCucuuguucgauggcacu...............................................................................................................................................................................   15     1     LT-1
......................uggaucuuguucgaCggcacu...............................................................................................................................................................................   1      1     LT-1
......................uggaucuuAuucgauggcacu...............................................................................................................................................................................   1      1     JCK-3
......................uggaucuuAuucgauggcacu...............................................................................................................................................................................   1      1     LT-3
......................uggaucuuguucgauggcCcu...............................................................................................................................................................................   3      1     LT-3
......................uggCucuuguucgauggcacu...............................................................................................................................................................................   12     1     LCK-3
......................uggaucuuguucgauggcaUu...............................................................................................................................................................................   23     1     LCK-1
......................uggaucuuguucgauggUacu...............................................................................................................................................................................   2      1     JCK-1
......................uggaucuuguucgauggcacG...............................................................................................................................................................................   1      1     LCK-1
......................uggaucCuguucgauggcacu...............................................................................................................................................................................   1      1     LCK-3
......................uggaucuuguucgauggcUcu...............................................................................................................................................................................   1      1     LT-3
......................uggaucuuguucgauggcCcu...............................................................................................................................................................................   2      1     LCK-2
......................uggaucuuguucgauggcaUu...............................................................................................................................................................................   8      1     LT-2
......................uggaucuuguucgauggcacN...............................................................................................................................................................................   1      1     JT-2
......................uggaucuuguucgauggcacC...............................................................................................................................................................................   11     1     LCK-3
......................uggaucuCguucgauggcacu...............................................................................................................................................................................   1      1     JCK-1
......................uggaucuugCucgauggcacu...............................................................................................................................................................................   2      1     JCK-2
......................uggaucuuguucgaugAcacu...............................................................................................................................................................................   4      1     JCK-3
......................uggaucuuguucgaCggcacu...............................................................................................................................................................................   1      1     LCK-2
......................ugAaucuuguucgauggcacu...............................................................................................................................................................................   2      1     LT-3
......................uggNucuuguucgauggcacu...............................................................................................................................................................................   1      1     LCK-1
......................uggaucCuguucgauggcacu...............................................................................................................................................................................   3      1     JCK-1
......................Cggaucuuguucgauggcacu...............................................................................................................................................................................   1      1     JCK-3
......................uggaCcuuguucgauggcacu...............................................................................................................................................................................   2      1     JCK-2
......................uggaucuuguucgaugAcacu...............................................................................................................................................................................   3      1     LCK-3
......................uggaucuuguucgaugAcacu...............................................................................................................................................................................   4      1     JCK-1
......................uUgaucuuguucgauggcacu...............................................................................................................................................................................   3      1     JCK-2
......................uggaucuuguuGgauggcacu...............................................................................................................................................................................   2      1     JCK-1
......................uggaucuuguucgCuggcacu...............................................................................................................................................................................   20     1     LT-2
......................uAgaucuuguucgauggcacu...............................................................................................................................................................................   1      1     JCK-1
......................Cggaucuuguucgauggcacu...............................................................................................................................................................................   1      1     LT-1
......................uggCucuuguucgauggcacu...............................................................................................................................................................................   24     1     JCK-1
......................uggaucuuguucgauggcacu...............................................................................................................................................................................   5446   0     LT-2
......................uggaucuuguucgCuggcacu...............................................................................................................................................................................   1      1     JT-2
......................Cggaucuuguucgauggcacu...............................................................................................................................................................................   1      1     JT-1
......................uUgaucuuguucgauggcacu...............................................................................................................................................................................   2      1     LT-2
......................ugAaucuuguucgauggcacu...............................................................................................................................................................................   3      1     JCK-3
......................uggaucuuguucgauAgcacu...............................................................................................................................................................................   26     1     JCK-2
......................uNgaucuuguucgauggcacu...............................................................................................................................................................................   10     1     JCK-1
......................uggaucCuguucgauggcacu...............................................................................................................................................................................   4      1     JCK-3
......................ugAaucuuguucgauggcacu...............................................................................................................................................................................   4      1     LT-2
......................uAgaucuuguucgauggcacu...............................................................................................................................................................................   4      1     LT-2
......................uggaucuuguCcgauggcacu...............................................................................................................................................................................   2      1     JCK-1
......................uggaucuuguucgauggcacA...............................................................................................................................................................................   61     1     LT-3
......................uggaucuuguucgauAgcacu...............................................................................................................................................................................   11     1     JCK-3
......................uggaucCuguucgauggcacu...............................................................................................................................................................................   6      1     JCK-2
......................uggaucuuguucgauggcGcu...............................................................................................................................................................................   1      1     LT-2
......................uggaucuuguuUgauggcacu...............................................................................................................................................................................   1      1     LCK-2
......................uggaucuuguucgauggcacG...............................................................................................................................................................................   2      1     LT-2
......................uggaucuuNuucgauggcacu...............................................................................................................................................................................   1      1     JCK-3
......................uggaucuuAuucgauggcacu...............................................................................................................................................................................   2      1     LCK-3
......................uggaucuuguucgCuggcacu...............................................................................................................................................................................   10     1     LT-1
......................uggaucuuguucgauggcacu...............................................................................................................................................................................   6201   0     LT-1
......................uggaucuuguucgauggcacC...............................................................................................................................................................................   7      1     JCK-2
```

```
                      Mature                                                        Star
uccaucuuguucguucacucucuggaucuuguucgauggcacuccccucuuauccaaucuuucucucucucuuaugaugcgaguggaagugccaugaacgugaucccagaaugugcuuuccaacaaguuacaccuacuuaauaucuacuucuuuuuucuuuuuucuuuuuucuuccauuacuauuauuaaggaugguuucuuuuucugguucugauuuuuauuuacuuauuuauuuauuuguuuuuugugu
......................ugCaucuuguucgauggcacu...............................................................................................................................................................................  1      1     LT-2
......................uggaucuuguCcgauggcacu...............................................................................................................................................................................  3      1     JCK-2
......................ugAaucuuguucgauggcacu...............................................................................................................................................................................  3      1     LCK-3
......................uAgaucuuguucgauggcacu...............................................................................................................................................................................  2      1     JCK-3
......................uAgaucuuguucgauggcacu...............................................................................................................................................................................  1      1     LT-1
......................uggauNuuguucgauggcacu...............................................................................................................................................................................  3      1     LCK-1
......................Cggaucuuguucgauggcacu...............................................................................................................................................................................  1      1     LT-3
......................Cggaucuuguucgauggcacu...............................................................................................................................................................................  4      1     JCK-2
......................uggaucuuguucgauggcaAu...............................................................................................................................................................................  1      1     JCK-3
......................uggaucuuguucgauggAacu...............................................................................................................................................................................  1      1     LCK-3
......................uggGucuuguucgauggcacu...............................................................................................................................................................................  1      1     LT-2
......................uggaucuuguucgauggcaAu...............................................................................................................................................................................  1      1     LCK-3
......................Gggaucuuguucgauggcacu...............................................................................................................................................................................  1      1     LT-3
......................uggaucuuguucgNuggcacu...............................................................................................................................................................................  12     1     LCK-1
......................uggaucuuguucgCuggcacu...............................................................................................................................................................................  2      1     JT-3
......................uggaucuuguucgauggcaAu...............................................................................................................................................................................  2      1     LT-1
......................uggaucuuguucUauggcacu...............................................................................................................................................................................  1      1     JCK-2
......................uggaucuuguucgauggcCcu...............................................................................................................................................................................  6      1     JCK-3
......................uggaucuuguucgauggcacC...............................................................................................................................................................................  15     1     LT-1
......................uNgaucuuguucgauggcacu...............................................................................................................................................................................  1      1     LCK-3
......................uggaucuCguucgauggcacu...............................................................................................................................................................................  1      1     LT-1
......................uggaucuugCucgauggcacu...............................................................................................................................................................................  1      1     LT-1
......................uggaucuuguucCauggcacu...............................................................................................................................................................................  1      1     JCK-3
......................uggaucCuguucgauggcacu...............................................................................................................................................................................  1      1     LT-3
......................uggaucuuguucgauggcacA...............................................................................................................................................................................  30     1     JCK-1
......................uggaucuuUuucgauggcacu...............................................................................................................................................................................  1      1     LT-1
......................uggaucuuguucgauggcaAu...............................................................................................................................................................................  3      1     JCK-1
......................uggaucuuguucgauggcacu...............................................................................................................................................................................  712    0     JT-1
......................uggaucuuguucgauAgcacu...............................................................................................................................................................................  2      1     LT-1
......................uggCucuuguucgauggcacu...............................................................................................................................................................................  53     1     LT-2
......................uggaucuuguuUgauggcacu...............................................................................................................................................................................  2      1     LT-1
......................uggaucuuguucAauggcacu...............................................................................................................................................................................  1      1     LCK-3
......................uggaucuuguucgauggcacu...............................................................................................................................................................................  7444   0     LT-3
......................uggaucuuguucgauggcCcu...............................................................................................................................................................................  6      1     LT-1
......................uggaucuuguucgauggGacu...............................................................................................................................................................................  4      1     JCK-1
......................uggaucuuguucgauggcaUu...............................................................................................................................................................................  8      1     JCK-3
......................uggaucuuguucgauggcacC...............................................................................................................................................................................  12     1     JCK-3
......................uggaucuuguucgauggcaUu...............................................................................................................................................................................  10     1     JCK-1
......................uggaucuCguucgauggcacu...............................................................................................................................................................................  2      1     JCK-2
......................ugAaucuuguucgauggcacu...............................................................................................................................................................................  8      1     JCK-2
......................uggaucuuguucgauggcacu...............................................................................................................................................................................  181    0     JT-2
......................uggaucuuguucgauggcaNu...............................................................................................................................................................................  8      1     JCK-1
......................uggaucuuguucgauggcNcu...............................................................................................................................................................................  6      1     JCK-1
......................Cggaucuuguucgauggcacu...............................................................................................................................................................................  4      1     LCK-3
......................uggaucuuguucgGuggcacu...............................................................................................................................................................................  1      1     JCK-1
......................uggaucuuguucgCuggcacu...............................................................................................................................................................................  33     1     JCK-2
......................uggaucuuguucgauggcacG...............................................................................................................................................................................  6      1     JCK-3
......................uggaucuuguucgCuggcacu...............................................................................................................................................................................  11     1     LCK-3
......................ugAaucuuguucgauggcacu...............................................................................................................................................................................  1      1     JCK-1
......................ugNaucuuguucgauggcacu...............................................................................................................................................................................  8      1     LT-2
......................uggauUuguucgauggcacu...............................................................................................................................................................................  1      1     LT-2
......................uggaucuuguucgCuggcacu...............................................................................................................................................................................  4      1     JT-1
......................uggaucuuguucgauggcacC...............................................................................................................................................................................  7      1     LT-2
......................uggaucuuguucgauggcaUu...............................................................................................................................................................................  14     1     LT-3
......................uggaucuuguucgauggcCcu...............................................................................................................................................................................  2      1     LCK-1
......................uggaucuuguucgauggcacC...............................................................................................................................................................................  3      1     JCK-1
......................uggaucuuguucgauggcacG...............................................................................................................................................................................  9      1     LT-3
......................uggaucuuguucgauggcacu...............................................................................................................................................................................  12438  0     JCK-3
......................uggaucuuguucgauggcacu...............................................................................................................................................................................  9876   0     JCK-1
......................uggaucuuguucgauggcaGu...............................................................................................................................................................................  1      1     JCK-1
......................uggaucuuguucgauggcaUu...............................................................................................................................................................................  12     1     JCK-2
......................uggaucuuguucgauggNacu...............................................................................................................................................................................  1      1     JCK-3
......................uggaucuuguucgauggcaUu...............................................................................................................................................................................  21     1     LCK-2
......................uggaucuuguucgaugUcacu...............................................................................................................................................................................  2      1     JCK-2
......................uggaucuuguucgauggcGcu...............................................................................................................................................................................  1      1     JCK-2
......................uggaucuuguucgGuggcacu...............................................................................................................................................................................  1      1     JCK-3
......................uggaucuuguucgCuggcacu...............................................................................................................................................................................  12     1     JCK-1
......................uggCucuuguucgauggcacu...............................................................................................................................................................................  25     1     LT-3
......................uggaucuuguucgaugAcacu...............................................................................................................................................................................  9      1     JCK-2
......................uggaucuuguucgauggcacu...............................................................................................................................................................................  288    0     JT-3
```

```
                      Mature                                                                Star
uccaucuuguucguucacucucuggaucuuguucgauggcacuccccucuuauccaaucuuucucucucucuuaugaugcgaguggaagugccaugaacgugauccagaaugugcuuuccaacaaguuacaccuacuuaauaucuacuucuuuuuucuuuuuucuuuuuucuuccauuacuauuauuaaggaugguuucuuuuucugguucugauuuuuauuuacuuauuuauuuauuuguuuuuugugu
......................uggaucAuguucgauggcacu...............................................................................................................................................................................   2      1   JCK-2
......................uAgaucuuguucgauggcacu...............................................................................................................................................................................   4      1   LT-3
......................uggaucuuguucgCuggcacu...............................................................................................................................................................................   18     1   LT-3
......................uggaucuuguucgauggUacu...............................................................................................................................................................................   1      1   LT-3
......................uggaucuuguucgUuggcacu...............................................................................................................................................................................   1      1   LT-2
......................uggaucuuguucgauggcacG...............................................................................................................................................................................   1      1   LCK-3
......................Gggaucuuguucgauggcacu...............................................................................................................................................................................   2      1   JCK-2
......................uggaucuuguucgauggcCcu...............................................................................................................................................................................   12     1   LT-2
......................uggaucuuguucgCuggcacu...............................................................................................................................................................................   1      1   LCK-1
......................uggaucuuAuucgauggcacu...............................................................................................................................................................................   3      1   LT-2
......................uggaucuuguucgaGggcacu...............................................................................................................................................................................   2      1   LCK-1
......................uggaucuuguucUauggcacu...............................................................................................................................................................................   4      1   JCK-1
......................ugAaucuuguucgauggcacu...............................................................................................................................................................................   3      1   LT-1
......................uggaucuuguucgauggcacG...............................................................................................................................................................................   7      1   JCK-2
......................uggaucuuguucgauggAacu...............................................................................................................................................................................   1      1   JCK-1
......................uggaucuuguuUgauggcacu...............................................................................................................................................................................   8      1   JCK-2
......................uggaucuuguucAauggcacu...............................................................................................................................................................................   2      1   JCK-3
......................uNgaucuuguucgauggcacu...............................................................................................................................................................................   18     1   JCK-2
......................uggaucuuAuucgauggcacu...............................................................................................................................................................................   1      1   JCK-2
......................uggCucuuguucgauggcacu...............................................................................................................................................................................   37     1   JCK-2
......................uggaCcuuguucgauggcacu...............................................................................................................................................................................   1      1   LCK-1
......................uggaucuuguucgauggcCcu...............................................................................................................................................................................   1      1   JT-1
......................uggCucuuguucgauggcacu...............................................................................................................................................................................   22     1   JCK-3
......................uggaucuuguucgauggcacG...............................................................................................................................................................................   7      1   JCK-1
......................uggGucuuguucgauggcacu...............................................................................................................................................................................   5      1   JCK-1
......................uggaucuuguucgaCggcacu...............................................................................................................................................................................   1      1   LT-2
......................uggaucuuguucgauggcaUu...............................................................................................................................................................................   10     1   LT-1
......................uggaucuuguucgauggcacu...............................................................................................................................................................................   1998   0   LCK-1
......................uggaCcuuguucgauggcacu...............................................................................................................................................................................   1      1   LCK-2
......................uggaucuuguucgauggUacu...............................................................................................................................................................................   5      1   JCK-3
......................uggaucuuguucgaCggcacu...............................................................................................................................................................................   10     1   JCK-3
......................uggaucuuguCcgauggcacu...............................................................................................................................................................................   1      1   LT-2
......................uggaucuuguucAauggcacu...............................................................................................................................................................................   3      1   LT-3
......................Cggaucuuguucgauggcacu...............................................................................................................................................................................   1      1   LT-2
......................uggaucuuguucgauggcCcu...............................................................................................................................................................................   5      1   LCK-3
......................uggaucuuguucgauggcUcu...............................................................................................................................................................................   1      1   LT-1
......................uggCucuuguucgauggcacu...............................................................................................................................................................................   6      1   LCK-1
......................uggaucuuguucgaugUcacu...............................................................................................................................................................................   1      1   JCK-1
......................uggaucuuguucgaugAcacu...............................................................................................................................................................................   1      1   JT-3
......................uggaucuuguucgaCggcacu...............................................................................................................................................................................   2      1   JCK-1
......................uggaucuuguucgauggcacu...............................................................................................................................................................................   17137  0   JCK-2
......................ugNaucuuguucgauggcacu...............................................................................................................................................................................   1      1   LCK-1
......................uggaucuuguucAauggcacu...............................................................................................................................................................................   1      1   LT-1
......................uggaucuuguucgauAgcacu...............................................................................................................................................................................   2      1   LT-3
......................uNgaucuuguucgauggcacu...............................................................................................................................................................................   1      1   LCK-1
......................uggaucuuguucgauggcaAu...............................................................................................................................................................................   8      1   LT-2
......................uggaucuuguucgauggcCcu...............................................................................................................................................................................   10     1   JCK-1
......................uggaucuuguucgauggcacA...............................................................................................................................................................................   100    1   LCK-3
......................uggaucuuguucgCuggcacu...............................................................................................................................................................................   18     1   JCK-3
......................uggaucuuguucgauggcacA...............................................................................................................................................................................   25     1   JCK-3
......................uggaucuuguucgauggcGcu...............................................................................................................................................................................   1      1   LCK-3
......................uggaucuuguucgaugUcacu...............................................................................................................................................................................   1      1   LT-3
......................uggaucuuguucAauggcacu...............................................................................................................................................................................   3      1   JCK-1
......................uggaucuuguuUgauggcacu...............................................................................................................................................................................   9      1   JCK-3
......................uggaucuuguucgauggcGcu...............................................................................................................................................................................   1      1   JCK-3
......................uggaucuuguucgaCggcacu...............................................................................................................................................................................   5      1   JCK-2
......................uggaucuuguuNgauggcacu...............................................................................................................................................................................   21     1   JCK-1
......................Gggaucuuguucgauggcacu...............................................................................................................................................................................   1      1   LT-2
......................uggaucuuguucgauggcCcu...............................................................................................................................................................................   21     1   JCK-2
......................uggaucuuguucgaCggcacu...............................................................................................................................................................................   1      1   LCK-3
......................uggaucuuguucgauggcacA...............................................................................................................................................................................   28     1   LT-2
......................uggaucuuguucgauggcacu...............................................................................................................................................................................   6606   0   LCK-3
......................uggaucuuguuAgauggcacu...............................................................................................................................................................................   1      1   JCK-3
......................uggaucuuguucUauggcacu...............................................................................................................................................................................   4      1   JCK-3
......................uggaucuuguucgauggcacu...............................................................................................................................................................................   1957   0   LCK-2
......................uggaucuuguucgauggcacC...............................................................................................................................................................................   15     1   LT-3
......................uggaucuuUuucgauggcacu...............................................................................................................................................................................   3      1   JCK-3
......................uggaucuuguuAgauggcacu...............................................................................................................................................................................   2      1   JCK-1
......................uggaucuuguucUauggcacuc..............................................................................................................................................................................   2      1   LT-3
......................uggaucuuguucgaugCcacuc..............................................................................................................................................................................   1      1   JCK-1
```

```
                      Mature                                        Star
uccaucuuguucguucacucucuggaucuuguucgauggcacuccccucuuauccaaucuuucucucucucuuaugaugcgaguggaagugccaugaacgugaucCagaaugugcuuuccaacaaguuacaccuacuuaauaucuacuucuuuuuucuuuuuucuuuuuucuuccauuacuauuauuaaggaugguuucuuuuucugguucugauuuuuauuuacuuauuuauuuauuuguuuuuugugu
......................ugAaucuuguucgauggcacuc.............................................................................................................................................................................................................  2      1  LT-2
......................uggaucuuguucgauggcCcuc.............................................................................................................................................................................................................  11     1  LT-3
......................uggaucuuguucUauggcacuc.............................................................................................................................................................................................................  1      1  JCK-3
......................uggaucuuguucgauggcacuU.............................................................................................................................................................................................................  72     1  LT-2
......................uggaucuuguucgCuggcacuc.............................................................................................................................................................................................................  1      1  JT-1
......................ugAaucuuguucgauggcacuc.............................................................................................................................................................................................................  4      1  JCK-3
......................uggaucuugCucgauggcacuc.............................................................................................................................................................................................................  2      1  LT-2
......................uggaucuuguucgauggcacCc.............................................................................................................................................................................................................  3      1  JCK-1
......................uggaucuuAuucgauggcacuc.............................................................................................................................................................................................................  1      1  JCK-3
......................ugCaucuuguucgauggcacuc.............................................................................................................................................................................................................  1      1  LT-2
......................uggaucuuguucgauggUacuc.............................................................................................................................................................................................................  1      1  LT-1
......................uggauAuuguucgauggcacuc.............................................................................................................................................................................................................  1      1  JCK-3
......................uggaucuuguucgauggcacuA.............................................................................................................................................................................................................  17     1  LT-2
......................uggCucuuguucgauggcacuc.............................................................................................................................................................................................................  29     1  JCK-1
......................uggaucuuAuucgauggcacuc.............................................................................................................................................................................................................  2      1  LT-1
......................uAgaucuuguucgauggcacuc.............................................................................................................................................................................................................  1      1  LCK-3
......................uggaucuugCucgauggcacuc.............................................................................................................................................................................................................  2      1  JCK-2
......................uggaucuuguucgaugNcacuc.............................................................................................................................................................................................................  2      1  LT-2
......................uggaucuuguucgauggcaUuc.............................................................................................................................................................................................................  6      1  LT-3
......................uggaucuuguucgaCggcacuc.............................................................................................................................................................................................................  2      1  LT-2
......................ugAaucuuguucgauggcacuc.............................................................................................................................................................................................................  3      1  JCK-2
......................uggaucCuguucgauggcacuc.............................................................................................................................................................................................................  1      1  LT-1
......................uggaucuuguucgauggcacuc.............................................................................................................................................................................................................  19469  0  JCK-2
......................uUgaucuuguucgauggcacuc.............................................................................................................................................................................................................  3      1  JCK-2
......................uggaucuuguucgauggcacuN.............................................................................................................................................................................................................  1      1  LT-2
......................uUgaucuuguucgauggcacuc.............................................................................................................................................................................................................  3      1  LT-2
......................uggaucuuguucgauggcacGc.............................................................................................................................................................................................................  1      1  JCK-3
......................uggaucuuguucgauAgcacuc.............................................................................................................................................................................................................  42     1  JCK-2
......................uggaucuuguucgCuggcacuc.............................................................................................................................................................................................................  17     1  LT-1
......................uggCucuuguucgauggcacuc.............................................................................................................................................................................................................  3      1  LCK-2
......................uggaucuuguucgCuggcacuc.............................................................................................................................................................................................................  1      1  LCK-2
......................uggaucuuguAcgauggcacuc.............................................................................................................................................................................................................  1      1  JCK-1
......................uNgaucuuguucgauggcacuc.............................................................................................................................................................................................................  25     1  JCK-2
......................uggaucuuguucgauggcacuG.............................................................................................................................................................................................................  4      1  LT-2
......................Cggaucuuguucgauggcacuc.............................................................................................................................................................................................................  1      1  LT-3
......................uggaucuuAuucgauggcacuc.............................................................................................................................................................................................................  1      1  LCK-1
......................uggaucuuguucAauggcacuc.............................................................................................................................................................................................................  3      1  LT-2
......................uggaucuuguucgauggcacCc.............................................................................................................................................................................................................  2      1  JCK-2
......................uggauUuuguucgauggcacuc.............................................................................................................................................................................................................  10     1  JCK-2
......................uggaucCuguucgauggcacuc.............................................................................................................................................................................................................  5      1  JCK-2
......................uggaucuuUuucgauggcacuc.............................................................................................................................................................................................................  1      1  JCK-3
......................uggaucuuguucgaNggcacuc.............................................................................................................................................................................................................  1      1  LCK-1
......................uggaucuuguucgauggcCcuc.............................................................................................................................................................................................................  7      1  LT-2
......................uggCucuuguucgauggcacuc.............................................................................................................................................................................................................  22     1  JCK-3
......................uAgaucuuguucgauggcacuc.............................................................................................................................................................................................................  4      1  LT-3
......................uggaucuuguuAgauggcacuc.............................................................................................................................................................................................................  1      1  LT-3
......................uggaCcuuguucgauggcacuc.............................................................................................................................................................................................................  6      1  JCK-2
......................uggaucuuguucgauggcaNuc.............................................................................................................................................................................................................  9      1  JCK-1
......................uggaucuuguucgCuggcacuc.............................................................................................................................................................................................................  17     1  JCK-3
......................uggaucuuUuucgauggcacuc.............................................................................................................................................................................................................  1      1  LT-3
......................Gggaucuuguucgauggcacuc.............................................................................................................................................................................................................  1      1  JCK-2
......................uggaucuuguucgauggcCcuc.............................................................................................................................................................................................................  24     1  JCK-2
......................Cggaucuuguucgauggcacuc.............................................................................................................................................................................................................  1      1  LT-1
......................uggaucuuguucAauggcacuc.............................................................................................................................................................................................................  3      1  JCK-1
......................uggaucuuguucgauggcaUuc.............................................................................................................................................................................................................  21     1  JCK-3
......................uggaCcuuguucgauggcacuc.............................................................................................................................................................................................................  1      1  LCK-1
......................uggaucuuguucAauggcacuc.............................................................................................................................................................................................................  1      1  LT-1
......................uggCucuuguucgauggcacuc.............................................................................................................................................................................................................  1      1  LCK-1
......................uggaucuuguucgauggcCcuc.............................................................................................................................................................................................................  7      1  JCK-1
......................uggaucuuguucgauggcacuN.............................................................................................................................................................................................................  6      1  JCK-1
......................uggaucuuguucgauggcacAc.............................................................................................................................................................................................................  1      1  LT-3
......................uggGucuuguucgauggcacuc.............................................................................................................................................................................................................  1      1  JCK-2
......................uggaucuuguucgauggUacuc.............................................................................................................................................................................................................  2      1  LT-2
......................uggaucuuguucgCuggcacuc.............................................................................................................................................................................................................  27     1  LT-3
......................uAgaucuuguucgauggcacuc.............................................................................................................................................................................................................  1      1  LT-2
......................uggauAuuguucgauggcacuc.............................................................................................................................................................................................................  1      1  JCK-2
......................uggCucuuguucgauggcacuc.............................................................................................................................................................................................................  29     1  LT-3
......................uggauAuuguucgauggcacuc.............................................................................................................................................................................................................  1      1  LT-1
......................uggaucuuguucgaCggcacuc.............................................................................................................................................................................................................  2      1  JCK-2
......................uggaucuuguucgauggcacuc.............................................................................................................................................................................................................  3568   0  LCK-3
```

```
                      Mature                                                          Star
uccaucuuguucguucacucucuggaucuuguucgauggcacuccccucuuauccaaucuuucucucucucuuaugaugcgaguggaagugccaugaacgugauccagaaugugcuuuccaacaaguuacaccuacuuaauaucuacuucuuuuuucuuuuuucuuuuuucuuccauuacuauuauuaaggaugguuucuuuuucugguucugauuuuuauuuacuuauuuauuuauuuguuuuuugugu
......................uggaucuuguucAauggcacuc..............................................................................................................................................................................................................    8       1    JCK-2
......................uggaucuuguucgauggcacuc..............................................................................................................................................................................................................    8004    0    LT-3
......................uggaucuuguucgauggcacCc..............................................................................................................................................................................................................    2       1    LT-3
......................uggaucuuAuucgauggcacuc..............................................................................................................................................................................................................    1       1    JCK-1
......................uggaucuuguucgaugAcacuc..............................................................................................................................................................................................................    2       1    LT-3
......................uggaucuuguucgauggcacuU..............................................................................................................................................................................................................    82      1    JCK-1
......................uggaucuuguucgauggUacuc..............................................................................................................................................................................................................    1       1    LT-3
......................ugAaucuuguucgauggcacuc..............................................................................................................................................................................................................    1       1    LCK-1
......................uggaucuuguucgauggcaAuc..............................................................................................................................................................................................................    1       1    JCK-3
......................uggaucuuguGcgauggcacuc..............................................................................................................................................................................................................    5       1    JCK-1
......................uggaucuuguucgauggcacuc..............................................................................................................................................................................................................    356     0    JT-1
......................uggaucuuguucgauggcGcuc..............................................................................................................................................................................................................    3       1    LCK-3
......................uggaucuuguucgauggcacuU..............................................................................................................................................................................................................    147     1    LT-1
......................uggaucuuguucgauggcacCc..............................................................................................................................................................................................................    4       1    JCK-3
......................uggaucuuguucgCuggcacuc..............................................................................................................................................................................................................    4       1    LCK-3
......................uggaucuuguucgauggcCcuc..............................................................................................................................................................................................................    3       1    LCK-3
......................ugAaucuuguucgauggcacuc..............................................................................................................................................................................................................    2       1    LT-1
......................uggaucuuguucgauggcacuA..............................................................................................................................................................................................................    53      1    JCK-3
......................uggaucuuguucgNuggcacuc..............................................................................................................................................................................................................    2       1    LCK-1
......................uggaucuuguucgauggcaUuc..............................................................................................................................................................................................................    33      1    JCK-2
......................uggaucuuguucgauAgcacuc..............................................................................................................................................................................................................    1       1    LT-3
......................uggaucuuguucgauUgcacuc..............................................................................................................................................................................................................    3       1    JCK-3
......................uggGucuuguucgauggcacuc..............................................................................................................................................................................................................    1       1    LT-2
......................uggaucuuNuucgauggcacuc..............................................................................................................................................................................................................    2       1    JCK-3
......................uggaucuuguucgauggcNcuc..............................................................................................................................................................................................................    5       1    JCK-1
......................uggaucuuguucgauggcacuc..............................................................................................................................................................................................................    7340    0    LT-1
......................uggaucuuguucgauggcacuU..............................................................................................................................................................................................................    30      1    LCK-2
......................uggaucuuguucgaugAcacuc..............................................................................................................................................................................................................    2       1    JCK-2
......................uggaucuuguucgCuggcacuc..............................................................................................................................................................................................................    16      1    JCK-1
......................uggaucuuguucgauggAacuc..............................................................................................................................................................................................................    1       1    LT-1
......................uggGucuuguucgauggcacuc..............................................................................................................................................................................................................    3       1    JCK-1
......................uggaucuuguucgauggcacuc..............................................................................................................................................................................................................    143     0    JT-2
......................uggaucuuguucgCuggcacuc..............................................................................................................................................................................................................    10      1    LT-2
......................uggaucuuguucgauggcCcuc..............................................................................................................................................................................................................    2       1    LCK-1
......................uggaucuuguCcgauggcacuc..............................................................................................................................................................................................................    2       1    JCK-1
......................uggCucuuguucgauggcacuc..............................................................................................................................................................................................................    34      1    LT-2
......................uggaucuuguucgauggcacuc..............................................................................................................................................................................................................    3507    0    LT-2
......................uggaucuuguucgauggcGcuc..............................................................................................................................................................................................................    12      1    JT-1
......................uggaucuuguucgauggcacuA..............................................................................................................................................................................................................    44      1    LT-3
......................uggaucuuguucgauggGacuc..............................................................................................................................................................................................................    4       1    JCK-1
......................uggaCcuuguucgauggcacuc..............................................................................................................................................................................................................    1       1    JCK-3
......................uggaucuuUuucgauggcacuc..............................................................................................................................................................................................................    2       1    JCK-1
......................uggaucuuguucgauggcacuU..............................................................................................................................................................................................................    98      1    JCK-3
......................uggaucuuguucgauggcacGc..............................................................................................................................................................................................................    2       1    JCK-2
......................uggaucuuguucgauggcacuU..............................................................................................................................................................................................................    113     1    LCK-3
......................uggaucuuguucgauggcaAuc..............................................................................................................................................................................................................    1       1    LT-3
......................uggaucuuguucgauggcacuc..............................................................................................................................................................................................................    825     0    LCK-2
......................uggaucuugGucgauggcacuc..............................................................................................................................................................................................................    1       1    LT-1
......................uggaucuuguucgaugUcacuc..............................................................................................................................................................................................................    1       1    JCK-2
......................uggaucuuAuucgauggcacuc..............................................................................................................................................................................................................    4       1    JCK-2
......................uUgaucuuguucgauggcacuc..............................................................................................................................................................................................................    3       1    LT-3
......................uggaucuuUuucgauggcacuc..............................................................................................................................................................................................................    1       1    JCK-2
......................uggaucuCguucgauggcacuc..............................................................................................................................................................................................................    1       1    JCK-1
......................uggaucuuguucgauggcacNc..............................................................................................................................................................................................................    1       1    JCK-1
......................uAgaucuuguucgauggcacuc..............................................................................................................................................................................................................    5       1    LT-1
......................uggaucuuguucUauggcacuc..............................................................................................................................................................................................................    5       1    JCK-2
......................ugAaucuuguucgauggcacuc..............................................................................................................................................................................................................    3       1    JCK-1
......................uggaucuuguucgauggcacuU..............................................................................................................................................................................................................    121     1    LT-3
......................uggCucuuguucgauggcacuc..............................................................................................................................................................................................................    16      1    LT-1
......................uggaucuuguucUauggcacuc..............................................................................................................................................................................................................    3       1    LT-1
......................ugAaucuuguucgauggcacuc..............................................................................................................................................................................................................    1       1    LCK-3
......................uggaucuuguuNgauggcacuc..............................................................................................................................................................................................................    18      1    JCK-1
......................uggaucuuguuGgauggcacuc..............................................................................................................................................................................................................    2       1    JCK-1
......................uggaucuuguucgaugAcacuc..............................................................................................................................................................................................................    2       1    JCK-3
......................uggaucuuguucgauggUacuc..............................................................................................................................................................................................................    6       1    JCK-3
......................uggaucuuguuUgauggcacuc..............................................................................................................................................................................................................    10      1    JCK-2
......................uggaCcuuguucgauggcacuc..............................................................................................................................................................................................................    3       1    JCK-1
......................uggaucuuguucgauggcacAc..............................................................................................................................................................................................................    1       1    JCK-3
......................uggaucuuguuUgauggcacuc..............................................................................................................................................................................................................    1       1    LT-2
......................Cggaucuuguucgauggcacuc..............................................................................................................................................................................................................    2       1    JCK-2
```

```
                      Mature                                                          Star
uccaucuuguucguucacucucuggaucuuguucgauggcacuccccucuuauccaaucuuucucucucucuuaugaugcgaguggaagugccaugaacgugauccagaaugugcuuuccaacaaguuacaccuacuuaauaucuacuucuuuuuucuuuuuucuuuuuucuuccauuacuauuauuaaggaugguuucuuuuucugguucugauuuuuauuuacuuauuuauuuauuuguuuuuugugu
......................uggCucuuguucgauggcacuc..............................................................................................................................................................................................................  8      1  LCK-3
......................uggaucuuguucgauggcacuA..............................................................................................................................................................................................................  37     1  JCK-1
......................uAgaucuuguucgauggcacuc..............................................................................................................................................................................................................  4      1  JCK-1
......................uggaucuuguuUgauggcacuc..............................................................................................................................................................................................................  3      1  JCK-1
......................uggaucuuguucgaugAcacuc..............................................................................................................................................................................................................  6      1  JCK-1
......................uggaucuuguucgauggUacuc..............................................................................................................................................................................................................  2      1  JCK-1
......................uggaucuuguucgauggcCcuc..............................................................................................................................................................................................................  11     1  JCK-3
......................uggauUuuguucgauggcacuc..............................................................................................................................................................................................................  10     1  JCK-3
......................uggauUuuguucgauggcacuc..............................................................................................................................................................................................................  5      1  JCK-1
......................uggaucuuguucgauggcCcuc..............................................................................................................................................................................................................  6      1  LT-1
......................uggaucuuguucgauggcacCc..............................................................................................................................................................................................................  2      1  LT-1
......................uggaCcuuguucgauggcacuc..............................................................................................................................................................................................................  1      1  LT-3
......................uggaucGuguucgauggcacuc..............................................................................................................................................................................................................  1      1  LT-1
......................uggaucuuguucgauggcacCc..............................................................................................................................................................................................................  1      1  JT-1
......................uggaucuuguuUgauggcacuc..............................................................................................................................................................................................................  7      1  JCK-3
......................uggaucuCguucgauggcacuc..............................................................................................................................................................................................................  1      1  JCK-3
......................uggaucuuguucgaCggcacuc..............................................................................................................................................................................................................  1      1  LT-1
......................uggaucuuguucgaCggcacuc..............................................................................................................................................................................................................  1      1  JCK-1
......................uggaucuuguucgauggcacuU..............................................................................................................................................................................................................  19     1  LCK-1
......................uggaucuuguucgauggcacuc..............................................................................................................................................................................................................  785    0  LCK-1
......................uggaucuuguucAauggcacuc..............................................................................................................................................................................................................  3      1  LT-3
......................uggaucuuguucgauggUacuc..............................................................................................................................................................................................................  4      1  JCK-2
......................uggaucuuguucgauggcacuA..............................................................................................................................................................................................................  58     1  LCK-3
......................uggaucuuguucgCuggcacuc..............................................................................................................................................................................................................  33     1  JCK-2
......................uggaucuuguucgauggcacuA..............................................................................................................................................................................................................  38     1  LT-1
......................Cggaucuuguucgauggcacuc..............................................................................................................................................................................................................  5      1  JCK-3
......................uggaucNuguucgauggcacuc..............................................................................................................................................................................................................  1      1  JCK-3
......................uggaucuuguucgauggcacuA..............................................................................................................................................................................................................  72     1  JCK-2
......................uAgaucuuguucgauggcacuc..............................................................................................................................................................................................................  3      1  JCK-2
......................Nggaucuuguucgauggcacuc..............................................................................................................................................................................................................  1      1  JCK-3
......................uggaucuuguucgauAgcacuc..............................................................................................................................................................................................................  2      1  LT-1
......................uggaucuuguucgauCgcacuc..............................................................................................................................................................................................................  1      1  JCK-2
......................uAgaucuuguucgauggcacuc..............................................................................................................................................................................................................  3      1  JCK-3
......................uggaucuuguucgauggcaUuc..............................................................................................................................................................................................................  3      1  LT-1
......................Cggaucuuguucgauggcacuc..............................................................................................................................................................................................................  1      1  LT-2
......................uggaucuuguucgauggcaGuc..............................................................................................................................................................................................................  1      1  JCK-1
......................uggaucuuguucgauggcacAc..............................................................................................................................................................................................................  1      1  JCK-1
......................uggaucuuguucAauggcacuc..............................................................................................................................................................................................................  4      1  JCK-3
......................uNgaucuuguucgauggcacuc..............................................................................................................................................................................................................  7      1  JCK-3
......................Cggaucuuguucgauggcacuc..............................................................................................................................................................................................................  1      1  JCK-1
......................uggaucuuguucgaugUcacuc..............................................................................................................................................................................................................  2      1  JCK-3
......................ugAaucuuguucgauggcacuc..............................................................................................................................................................................................................  1      1  LT-3
......................uggaucuuguucgauggcacuc..............................................................................................................................................................................................................  11364  0  JCK-1
......................uggGucuuguucgauggcacuc..............................................................................................................................................................................................................  2      1  JCK-3
......................uggaucuuguucgauggcacAc..............................................................................................................................................................................................................  1      1  JCK-2
......................uNgaucuuguucgauggcacuc..............................................................................................................................................................................................................  15     1  JCK-1
......................uggaucuuguuUgauggcacuc..............................................................................................................................................................................................................  2      1  LT-1
......................uggaucuuguuNgauggcacuc..............................................................................................................................................................................................................  1      1  JT-1
......................uggaucuuguucgauggcaUuc..............................................................................................................................................................................................................  6      1  LT-2
......................uggaucCuguucgauggcacuc..............................................................................................................................................................................................................  1      1  JCK-1
......................uggaucuuguucgaCggcacuc..............................................................................................................................................................................................................  4      1  JCK-3
......................uggaucuuguucgauggcacuc..............................................................................................................................................................................................................  177    0  JT-3
......................uggaucuuguCcgauggcacuc..............................................................................................................................................................................................................  1      1  JCK-3
......................uggaucuuguucgauggcaUuc..............................................................................................................................................................................................................  19     1  JCK-1
......................uggaucuuguucgauggcacuU..............................................................................................................................................................................................................  147    1  JCK-2
......................uggaucuuguucgauggNacuc..............................................................................................................................................................................................................  2      1  JCK-3
......................uggCucuuguucgauggcacuc..............................................................................................................................................................................................................  1      1  JT-1
......................uggaucuuguucgauAgcacuc..............................................................................................................................................................................................................  26     1  JCK-3
......................uggaucuuguucgauggcacuG..............................................................................................................................................................................................................  8      1  JCK-1
......................uggNucuuguucgauggcacuc..............................................................................................................................................................................................................  6      1  JCK-1
......................uggaucuuguucgCuggcacuc..............................................................................................................................................................................................................  2      1  LCK-1
......................uggaucuuguucgauggcaUuc..............................................................................................................................................................................................................  4      1  LCK-3
......................uggaucuuguucgauAgcacuc..............................................................................................................................................................................................................  10     1  JCK-1
......................uggaucuuguucgauggcacuA..............................................................................................................................................................................................................  17     1  LCK-1
......................uggaucuugCucgauggcacuc..............................................................................................................................................................................................................  1      1  JCK-3
......................uggaucuuguucgauggcacuc..............................................................................................................................................................................................................  14328  0  JCK-3
......................uggaucuuguucgGuggcacuc..............................................................................................................................................................................................................  1      1  LT-2
......................uggaucuuguucgaugCcacuc..............................................................................................................................................................................................................  2      1  JCK-3
......................ugNaucuuguucgauggcacuc..............................................................................................................................................................................................................  10     1  LT-2
......................uggaucuuguucgauggcacuA..............................................................................................................................................................................................................  18     1  LCK-2
```

Mature **Star**

uccaucuuguucguucacucucuggaucuuguucgauggcacuccccucuuauccaaucuuucucucucucuuaugaugcgaguggaagugccaugaacgugauccagaaugugcuuuccaacaaguuacaccuacuuaauaucuacuucuuuuuucuuuuuucuuuuuucuuccauuacuauuauuaaggaugguuucuuuuucugguucugauuuuuauuuacuuauuuauuuauuuguuuuuugugu

uggCucuuguucgauggcacuc	30	1	JCK-2
uggaucuugCucgauggcacuc	2	1	LT-1
uggaucCuguucgauggcacuc	5	1	LCK-3
uggaucuuguucgauggcacucc	21	0	LT-3
uggaucuuguucgauggcacucc	12	0	LT-2
uggaucuuguucgauggcacucU	59	1	LT-1
uggaucuuguucgauggcacucA	56	1	LT-1
uggaucuuguucgauggcacucU	72	1	JCK-1
uggaucuuguucgauggcacucA	55	1	JCK-3
uggaucuuguucgauggcacucU	67	1	LT-2
uggaucuuguucgauggcacucc	18	0	JCK-3
uggaucuuguucgauggcacucA	41	1	JCK-1
uggaucuuguucgauggcacucG	1	1	LCK-3
uggaucuuguucgauggcacucA	33	1	LT-3
uggaucuuguucgauggcacucA	23	1	LCK-3
uggaucuuguucgauggcacucG	3	1	JCK-2
uggaucuuguucgauggcacucG	1	1	JCK-3
uggaucuuguucgauggcacucc	16	0	JCK-1
uggaucuuguucgauggcacucA	4	1	JT-2
uggaucuuguucgauggcacucA	70	1	JCK-2
uggaucuuguucgauggcacucU	89	1	JCK-3
uggaucuuguucgauggcacucc	7	0	LCK-3
uggaucuuguucgauggcacucA	6	1	JT-3
uggaucuuguucgauggcacucU	49	1	LT-3
uggaucuuguucgauggcacucU	125	1	JCK-2
uUgaucuuguucgauggcacucc	1	1	JCK-3
uggaucuuguucgauggcacucU	6	1	JT-3
uggaucuuguucgauggcacucU	12	1	JT-2
uggaucuuguucgauggcacucG	1	1	LT-3
uggaucuuguucgauggcacuAc	1	1	LT-1
uggaucuuguucgauggcacucU	10	1	JT-1
uggaucuuguucgauggcacucU	88	1	LCK-3
uggaucuuguucgauggcacucG	1	1	LT-1
uggaucuuguucgauggcacucA	28	1	LT-2
uggaucuuguucgauggcacuUc	2	1	LT-3
uggaucuuguucgauggcacucc	28	0	LT-1
uggaucuuguucgauggcacuUc	2	1	LT-2
uggaucuuguucgauggcacucc	34	0	JCK-2
uggaucuuguucgauggcacuAc	1	1	LT-2
uggaucuuguucgauggcacuUc	1	1	JCK-1
uggaucuuguucgauggcacuccU	5	1	LCK-3
uggaucuuguucgauggcacuccU	3	1	LT-3
uggaucuuguucgauggcacucAc	4	1	LT-1
uggaucuuguucgauggcacucAc	7	1	LCK-3
uggaucuuguucgauggcacuccc	2	0	LT-1
uggaucuuguucgauggcacuccU	4	1	LT-1
uggaucuuguucgauggcacucGc	2	1	LT-2
uggaucuuguucgauggcacucGc	3	1	LT-1
uggaucuuguucgauggcacuccc	4	0	LCK-3
uggaucuuguucgauggcacuccU	10	1	LT-2
uggaucuuguucgauggcacucUc	1	1	LT-2
uggaucuuguucgauggcacucAc	10	1	LT-2
uggaucuuguucgauggcacucccU	1	1	LT-1
uggaucuuguucgauggcacuccAc	4	1	LT-2
uggaucuuguucgauggcacuccAc	1	1	JCK-3
uggaucuuguucgauggcacuccAc	5	1	LCK-3
uggaucuuguucgauggcacuccAc	2	1	JCK-1
uggaucuuguucgauggcacucccU	1	1	LT-2
uggaucuuguucgauggcacuccAc	1	1	LT-1
uggaucuuguucgauggcacuccAc	2	1	JCK-2
uggaucuuguucgauggcacucccc	1	0	LT-3
uggaucuuguucgauggcacucUcc	4	1	LCK-3
uggaucuuguucgauggcacucUccu	1	1	LT-1
ggaucuuguucgauggca	5	0	JT-1
ggaucuuguucgauggcacu	3	0	JCK-3
ggaucuuguucgauggcacu	1	0	JCK-1
ggaucuuguucgauggcacu	3	0	LCK-3
ggaucuuguucgauggcacu	4	0	JCK-2
Ugaucuuguucgauggcacu	1	1	JCK-1
ggaucuuguucgauggcacu	10	0	LT-3

Mature **Star**

uccaucuuguucguucacucucuggaucuuguucgauggcacuccccucuuauccaaucuuucucucucucuuaugaugcgaguggaagugccaugaacgugauccagaaugugcuuuccaacaaguuacaccuacuuaauaucuacuucuuuuuucuuuuuucuuuuuucuuccauuacuauuauuaaggaugguuucuuuuucugguucugauuuuuauuuacuuauuuauuuauuuguuuuuugugu			
.......................ggaucuuguucgauggcacu	7	0	LT-1
.......................ggaucuuguucgauggcacu	6	0	LT-2
.......................ggaucuuguucgauggcacuc	2	0	LCK-3
.......................ggaucuuguucgauggcacuc	3	0	LT-3
.......................ggaucuuguucgauggcacuc	2	0	LT-1
.......................ggaucuuguucgauggcacuc	6	0	JCK-1
.......................ggaucuuguucgauggcacuc	3	0	JCK-3
.......................ggaucuuguucgauggcacuc	2	0	JCK-2
.......................ggaucuuguucgauggcacucc	3	0	JCK-1
.......................ggaucuuguucgauggcacucc	1	0	JCK-3
.......................ggaucuuguucgauggcacucc	1	0	JCK-2
.......................ggaucuuguucgauggcacucc	1	0	LT-1
.......................ggaucuuguucgauggcacuccc	3	0	JCK-3
.......................ggaucuuguucgauggcacuccc	4	0	JCK-1
........................gaucuuguucgauggcacu	2	0	JCK-1
........................gaucuuguucgauggcacu	2	0	LT-2
........................gaucuuguucgauggcacu	1	0	JCK-3
........................gaucuuguucgauggcacuc	4	0	LT-2
........................gaucuuguucgauggcacuc	1	0	LT-3
........................gaucuuguucgauggcacuc	1	0	JCK-2
........................gaucuuguucgauggcacucc	12	0	JCK-2
........................gaucuuguucgauggcacucc	8	0	JCK-3
........................gaucuuguucgauggcacucc	1	0	LT-2
........................gaucuuguucgauggcacucc	1	0	LT-3
........................gaucuuguucgauggcacucc	5	0	JCK-1
........................Aaucuuguucgauggcacuccc	1	1	JCK-2
........................gaucuuguucgauggcacuccc	6	0	LT-3
........................gaucuuguucgauggcacuccc	56	0	JCK-2
........................gaucuuguucgauggcacuccc	5	0	LCK-3
........................gaucuuguucgauggcacuccU	1	1	JCK-3
........................gaucuuguucgauggcacuccc	37	0	JCK-3
........................gaucuuguucgauggcacuccc	24	0	JCK-1
........................gaucuuguucgauggcacuccc	2	0	LT-1
........................gaucuuguucgauggcacuccc	6	0	LT-2
.........................aucuuguucgauggcacu	1	0	LT-2
.........................aucuuguucgauggcacuc	1	0	JCK-3
.........................aucuuguucgauggcacuc	1	0	JCK-1
.........................aucuuguucgauggcacuc	3	0	LT-3
.........................aucuuguucgauggcacucc	1	0	JCK-2
.........................aucuuguucgauggcacucc	2	0	LT-2
.........................aucuuguucgauggcacucc	3	0	LT-3
.........................aucuuguucgauggcacucc	1	0	JCK-3
.........................aucuuguucgauggcacucU	1	1	LT-1
.........................aucuuguucgauggcacucU	1	1	JCK-3
.........................aucuuguucgauggcacucU	2	1	JCK-2
.........................aucuuguucgauggcacucc	1	0	JCK-1
.........................aucuuguucgauggcacuccc	5	0	JCK-2
.........................aucCuguucgauggcacuccc	1	1	LT-3
.........................aucuuguucgauggcacuccc	1	0	LT-3
.........................aucuuguucgauggcacuccc	2	0	JCK-3
.........................aucuuguucgauggcacuccc	1	0	LT-1
.........................aucuuguucgauggcacucccc	2	0	LT-3
..........................ucuuguucgauggcacuc	1	0	JCK-3
..........................ucuuguucgauggcacucc	4	0	LCK-3
..........................ucuuguucgauggcacuccc	2	0	LT-3
................................ucgauggcacucccucuuauc	1	0	LT-3
....cuuauccaaucuuucucucucucuuaug	1	0	JCK-2
....cuuauccaaucuuucucucucucuuaug	2	0	JCK-1
....uggaagugccaugaacgugauc	2	0	LT-2
....aagugccaugaacgugUu	2	1	LT-3
....aagugccaugaacgugauc	4	0	JCK-1
....aagugccaugaacgugauc	7	0	LCK-3
....aagugccaugaacgugauc	23	0	LCK-1
....aagugccaugaacgugauc	14	0	JCK-3
....aagugccaugaacgugauc	24	0	LT-3
....aagugccaugaacgugauc	28	0	LT-1
....aaAugccaugaacgugauc	2	1	LT-2
....aagugccaugaacgugauU	1	1	LT-2
....aagugccaugaacgugauc	17	0	LT-2
....aagugccaugaacgugauc	34	0	LCK-2

Mature **Star**

uccaucuuguucguucacucucuggaucuuguucgauggcacuccccucuuauccaaucuuucucucucucuuaugaugcgaguggaagugccaugaacgugaucccagaaugugcuuuccaacaaguuacaccuacuuaauaucuacuucuuuuuucuuuuuucuuuuuucuuccauuacuauuauuaaggaugguuucuuuuucugguucugauuuuuauuuacuuauuuauuuauuuguuuuuugugu

aagugccaugaacgugauc	23	0	JCK-2
aagugccaugaacgugaucc	8	0	LT-2
aagugccaugaacgugaucc	13	0	LT-3
aagugccaugaacgugaucc	2	0	JCK-1
aagugccaugaacgugaucc	3	0	JCK-2
aagugccaugaacgugaucU	5	1	LCK-3
aagugccaugaacgugaucc	5	0	LT-1
aagugccaugaacgugaucc	6	0	JCK-3
aagugccaugaacgugaucU	1	1	LT-1
aagugccaugaacgugaucc	11	0	LCK-3
aagugccaugaacgugaucU	4	1	LT-2
aagugccaugaacgugaucca	3	0	JCK-3
aagugccaugaacgugaucca	2	0	JCK-1
aagugccaugaacgugaucca	1	0	LT-3
aagugccaugaacgugaucca	4	0	JCK-2
agugccaugaacgugauc	1	0	JCK-3
agugccaugaacCugauc	1	1	JCK-2
agugccaugaacgugauc	1	0	JCK-2
gugccaugaacgugaucca	1	0	LT-1
gugccaugaacgugauccag	1	0	JCK-3
gugccaugaacCugauccag	1	1	LT-2
gugccaugaacgugauccaga	19	0	LCK-1
gugccaugaacNugauccaga	1	1	JT-1
gugccaugaacgugauccaga	3	0	LT-1
gugccaugaacgugauccaga	2	0	LT-2
gugccaugaacCugauccaga	1	1	JCK-1
gugccaugaacgugauccaga	5	0	LCK-3
gugccaugaacNugauccaga	7	1	JCK-1
gugccCugaacgugauccaga	1	1	JCK-1
gugccaugaacgugauccaga	14	0	LT-3
gugccaugaacgugauccaga	16	0	JCK-1
gugccaugaacgugauccaga	8	0	LCK-2
gugccaugaacgugauccaga	26	0	JCK-3
gugccaugaacgugauccaga	45	0	JCK-2
ugccaugaacgugauccag	2	0	JCK-1
ugccaugaacgugauccag	2	0	JCK-2
ugccaugaacgugauccaga	11	0	JCK-3
ugccaugaacgugauccaga	3	0	JT-2
ugccaugaacgugNuccaga	1	1	LCK-1
ugccaugaacgugauccaga	14	0	LCK-2
ugccaugCacgugauccaga	1	1	LCK-3
ugccaugaacgugauccaga	4	0	JT-3
ugccaugaacgugauccaga	20	0	LCK-1
ugccaugaacgugauccaga	5	0	LT-1
uNccaugaacgugauccaga	1	1	LCK-1
ugccaugaacgugauccaga	12	0	JCK-2
ugccaugaacgugauccagU	1	1	JCK-1
ugccaugaacgugauccaga	14	0	JCK-1
ugccaugaacgugauccaga	11	0	LT-2
ugccaugaacgugauccaga	14	0	LCK-3
ugccaugaacgugauccaga	3	0	LT-3
aacgugauccagaaugugcuuuc	6	0	LCK-3
uuuucuuuuuuAuuccauuacu	1	1	JT-3
uuuucuuuuuuAuuccauuacu	2	1	JT-2
ucugguucugauuuuuauuuacuuauu	1	0	JCK-1
ucugguucugauuuuuauuuacuuauu	1	0	JCK-3
ucugguucugauuuuuauuuacuuauu	1	0	JCK-2
